# Supplementary material for: Pilot Study to Develop and Test Palliative Care Quality Indicators for Nursing Homes
Source: Int J Environ Res Public Health. 2021 Jan 19;18(2):829. doi: 10.3390/ijerph18020829 (PMC7835963; doi:10.3390/ijerph18020829)
Supplement: Supplementary file 1 [file ijerph-18-00829-s001.pdf]

Table S1: list of quality indicators with numerators, denominators and questions

Domain: Physical aspects of care					
N°	Short title	Numerator and denominator of the indicator	Respondent	Question	Evidence
PC-1	Being in pain	Numerator: Number of residents with a pain score of 3 or more in the last three days<br>Denominator: all residents who stayed longer than 1 month in the nursing home, with whom the indicator was measured	Residents	Please tick the box that best describes how pain has affected you over the past 3 days:<br>• not at all,<br>• slightly,<br>• moderately,<br>• severely,<br>• overwhelmingly	Indicator developed by researchers and expert panel<br><br>Measurement question from "IPOS-5"
**Domain: Psychological, social or spiritual aspects of care**					
N°	Short title	Numerator and denominator of the indicator	Respondent	Question	Source
PC-2	Feeling worried or anxious, or a burden	Numerator: Number of residents who indicate they were most of the times or always feeling worried or anxious, or a burden to others<br>Denominator: all residents who stayed longer than 1 month in the nursing home, with whom the indicator was measured	Residents	Are you feeling worried or anxious, or a burden to others?<br>• always<br>• often<br>• sometimes<br>• rarely	Indicator developed by researchers and expert panel<br><br>Measurement question adapted from "ICECAP-SCM"
PC-3	Being around people who care about you	Numerator: Number of residents who indicate that they were most of the times or always able to be around people who cared about them<br>Denominator: all residents who stayed longer than 1 month in the nursing home, with whom the indicator was measured	Residents	If I want to, I am able to be with people who care about me:<br>• most of the time<br>• some of the time<br>• only a little of the time<br>• never	Indicator developed by researchers and expert panel<br><br>Measurement question from "ICECAP-SCM"
**Domain: Autonomy and dignity**					
N°	Short title	Numerator and denominator of the indicator	Respondent	Question	Source
PC-4	Personal wishes and beliefs respected	Numerator: Number of residents who indicate that their caregivers most of the times or always respecting their personal wishes and beliefs<br>Denominator: all residents who stayed longer than 1 month in the nursing home, with whom the indicator was measured	Residents	Do your professional carers take your personal wishes and beliefs into account?<br>• most of the time<br>• some of the time<br>• only a little of the time<br>• never	Indicator originally from Claessen et al: ''extent to which patients experience respect for their autonomy''

					Measurement question from "QPAC study"
PC-5	Decisions about life and care	Numerator: Number of residents who indicate that they most of the times or always can make their own decisions about their life and care<br>Denominator: all residents who stayed longer than 1 month in the nursing home, with whom the indicator was measured	Residents	I am able to make decisions that I need to make about my life and care:<br>• most of the time<br>• some of the time<br>• only a little of the time<br>• never	Indicator developed by researchers and expert panel<br><br>Measurement question from "ICECAP-SCM"
PC-6	Treated with respect	Numerator: Number of residents who indicate that they most of the times or always were treated with respect<br>Denominator: all residents who stayed longer than 1 month in the nursing home, with whom the indicator was measured	Residents	Do you feel treated with respect? (respectfully addressed, respect for beliefs, being yourself, having privacy):<br><br>• most of the time<br>• some of the time<br>• only a little of the time<br>• never	Indicator developed by researchers and expert panel<br><br>Measurement question adapted from "ICECAP-SCM"
**Domain: Communication with residents**					
<u>N°</u>	<u>Short title</u>	<u>Numerator and denominator of the indicator</u>	<u>Respondent</u>	<u>Question</u>	<u>Source</u>
ACP-1	Information comprehensible and not contradictory	Numerator: Number of residents who indicate that they most of the times or always receive comprehensible information and almost never of never contradictory information<br>Denominator: all residents who stayed longer than 1 month in the nursing home, with whom the indicator was measured	Residents	Do your professional carers explain things to you understandably?<br>• Never<br>• Sometimes<br>• Usually<br>• Always<br><br>Do your professional carers give you conflicting information?<br>• Never<br>• Sometimes<br>• Usually<br>• Always	Indicator originally from Claessen et al: ''extent to which patients indicate that they receive understandable explanations'' and ''extent to which patients indicate that they receive contradictory information''<br><br>Measurement question from "QPAC study"

**Domain: Care planning**					
N°	Short title	Numerator and denominator of the indicator	Respondent	Question	Source
ACP-2	Conversation with family	Numerator: Number of residents for whom the next-of-kin indicates that more than once a conversation took place with the caregivers, the next-of-kin and, when possible, the resident<br>Denominator: all residents who stayed longer than 1 month in the nursing home, for whom the indicator was measured	Next-of-kin	How often did the professional carers organize a meeting, to which you and your relative were invited?<br>□ More than once<br>□ Once<br>□ Never	Indicator adapted from QPAC study: "a conversation about their care preferences took place between the professional caregivers and family carers in the first week after admission or start of palliative care"<br><br>Measurement question adapted from "QPAC study"
ACP-3	Knowledge about care goals and life wishes	Numerator: Number of residents for whom their professional caregiver indicates that they have knowledge about the residents' care goals and life wishes.<br>Denominator: all residents who stayed longer than 1 month in the nursing home, for whom the indicator was measured	Professional caregiver	Do you know the care goals and life wishes of this resident?<br>• Yes<br>• No	Indicator developed by researchers and expert panel<br><br>Measurement question developed by researchers
ACP-4	Encouraging ACP	Numerator: Number of residents for whom their professional caregiver indicates that they often or very often encourage residents and their next-of-kins to involve in advance care planning.<br>Denominator: all residents who stayed longer than 1 month in the nursing home, for whom the indicator was measured	Professional caregiver	I encourage residents and families to complete advanced care planning:<br>• Very much<br>• Much<br>• Neither much, nor little<br>• Little<br>• Very little	Indicator developed by researchers and expert panel<br><br>Measurement question from "PACE LTCF staff attitudes-communication and end-of-life care"
**Domain: care and communication for next-of-kin**					
N°	Short title	Numerator and denominator of the indicator	Respondent	Question	Source

ACP-5	Next-of-kin involved in decisions	Numerator: Number of next-of-kin who indicate that they often or very often felt involved in the decisions taken about the resident.<br>Denominator: all residents who stayed longer than 1 month in the nursing home, for whom the indicator was measured	Next-of-kin	I felt fully involved in all decision making of my relative:<br>• Very much<br>• Much<br>• Neither much, nor little<br>• Little<br>• Very little	Indicator developed by researchers and expert panel<br><br>Measurement question from "SWC-EOLD"
EOL-1	Information about approaching death	Numerator: umber of next-of-kin who indicate that they received the right amount of information on the approaching death of the resident.<br>Denominator: all residents who stayed longer than 1 month in the nursing home, for whom the indicator was measured	Next-of-kin	Did you get information about the impending death of your relative?<br>• less than necessary<br>• just the right amount<br>• more than necessary	Indicator of QPAC study "Family received the right amount of information about the patient's approaching death"<br><br>Measurement question of "QPAC study"<br><br>*Modified indicator from Miyashita et al: ''explanation to family of patient's impending death''*
PC-7	Attention for wishes and feelings of next-of-kin	Numerator: Number of next-of-kin who indicate that the professional caregivers had attention for their wishes and feelings.<br>Denominator: all residents who stayed longer than 1 month in the nursing home, for whom the indicator was measured	Next-of-kin	The health care team was sensitive to my needs and feeling:<br>• Very much<br>• Much<br>• Neither much, nor little<br>• Little<br>• Very little	Modified indicator from Claessen: ''Extent to which, according to the direct relatives, attention was paid to their own psychosocial and spiritual well-being''

					Measurement question from “SWC-EOLD” SWC-EOLD
EOL-2	Supported immediate after death	Numerator: Number of next-of-kin who indicate that they felt sufficiently supported by the professional caregivers immediate after the death of the resident.<br>Denominator: all residents who stayed longer than 1 month in the nursing home, for whom the indicator was measured	Next-of-kin	Did you feel supported by the professional carers immediately after the death of your relative?<br>• Yes<br>• No	Indicator from Claessen et al: ‘’Extent to which direct relatives felt supported by the caregivers immediately after the patient’s death,’’<br><br>Measurement question from “QPAC study”
**Domain: Communication among caregivers**					
N°	Short title	Numerator and denominator of the indicator	Respondent	Question	Source
PC-8	Information in resident file	Numerator: Number of residents for whom the professional caregiver finds sufficient information in the resident file when needed.<br>Denominator: all residents who stayed longer than 1 month in the nursing home, for whom the indicator was measured	Professional caregiver	Do you find the information on the resident in his or her file when you need it?<br>• Never<br>• Sometimes<br>• Usually<br>• Always	Indicator developed by researchers and expert panel<br><br>Measurement question developed by researchers
**Domain: care and circumstances surrounding death**					
N°	Short title	Numerator and denominator of the indicator	Respondent	Question	Source
EOL-3	Comfortable in last week of life	Numerator: Number of next-of-kin who indicate that many or a lot of measures were taken to make the resident comfortable in the last week of life.<br>Denominator: all residents who stayed longer than 1 month in the nursing home, for whom the indicator was measured	Next-of-kin	All measures were taken to keep my care recipient comfortable in the last week of life:<br>• Very much<br>• Much<br>• Neither much, nor little<br>• Little	Modified indicator from QPAC study: “symptom burden mostly or completely under control in the final week

				• Very little	of life"<br><br>Measurement question adapted from "SWC-EOLD"
EOL-4	Recognizing the approaching death	Numerator: Number of residents for whom the professional caregiver indicates they could recognize the approaching death well or very well by physical changes.<br>Denominator: all residents who stayed longer than 1 month in the nursing home, for whom the indicator was measured	Professional caregiver	I can recognize impending death (physical changes):<br>• Very good<br>• Good<br>• Neither good, nor bad<br>• Bad<br>• Very bad	Indicator developed by researchers and expert panel<br><br>Measurement question from "PACE LTCF staff attitudes-communication and end-of-life care"
EOL-5	Satisfied by care delivered	Numerator: Number of residents for whom the professional caregiver indicates they are satisfied with the care delivered to the resident.<br>Denominator: all residents who stayed longer than 1 month in the nursing home, for whom the indicator was measured	Professional caregiver	Are you satisfied by the care that you and your and your team members delivered to the resident?<br>• Very satisfied<br>• Satisfied<br>• Neither satisfied, nor dissatisfied<br>• Dissatisfied<br>• Very dissatisfied	Indicator developed by researchers and expert panel<br><br>Measurement question from "PACE LTCF staff attitudes-communication and end-of-life care"
EOL-6	Support by specialized palliative care	Numerator: Number of residents for whom the professional caregiver indicates a palliative care referent or specialized team was involved in the care for the resident.<br>Denominator: all residents who stayed longer than 1 month in the nursing home, for whom the indicator was measured	Professional caregiver	Was a specialized palliative care team involved in the care of this resident? (the specialized care team of the nursing home or an external team)<br>• Yes, the palliative care team of the nursing home<br>• Yes, an external palliative care team<br>• No	Indicator developed by researchers and expert panel<br><br>Measurement question developed by researchers
